# Medical interns' knowledge and training regarding urethral catheter insertion and insertion-related urethral injury in male patients

**DOI:** 10.1186/1472-6920-11-73

**Published:** 2011-09-27

**Authors:** Manuel Manalo, Marie Carmela M Lapitan, Brian S Buckley

**Affiliations:** 1 Division of Urology, Department of Surgery, Philippine General Hospital, University of the Philippines, Manila; 2 National Institutes of Health, University of the Philippines, Manila; 3 Department of Surgery, Philippine General Hospital, University of the Philippines, Manila

**Keywords:** urinary catheterization, education, medical, injuries, urethral

## Abstract

**Background:**

Improper catheterization can lead to urethral injury. Yet research from four continents suggests training of junior doctors in catheterization is insufficient. European research suggests a majority of catheterization related morbidities occur when the procedure is performed by interns.

**Methods:**

To assess the knowledge and practices of medical interns relating to urethral catheterization and iatrogenic urethral injury secondary to traumatic catheter insertion, a questionnaire survey was conducted of all first year medical interns at a tertiary national university hospital in the Philippines. The questionnaire contained 17 items covering 4 areas: methods of training in catheterization and level of experience; perceived adequacy of training; theoretical knowledge of catheterization; the mechanisms of catheter-related urethral injury.

**Results:**

225/240 interns (94%) completed the survey (130 (57.8%) female). 125 (55.6%) responded that they had adequate theoretical training and 150 (66.7%) adequate practical training. All had performed more than 10 catheterizations and 204 (90%) were supervised when they first performed catheterization. Despite relatively high levels of experience and confidence, deficits were identified in detailed knowledge of correct catheterization procedures and of risks associated with urethral injury.

**Conclusions:**

More thorough training of incoming medical interns in urinary catheterization may help to reduce the risk of complications and injury. Training should be universal and thought given to its timing within the curriculum. Training should include step by step instruction in the process, emphasis on history taking and awareness of factors associated with increased risk of urethral injury.

## Background

Improper catheterization can lead to urethral injury. Trauma can result from misjudged application of pressure during catheter insertion or from inflation of the balloon while still in the urethra. Although seldom life-threatening, iatrogenic urethral injury associated with catheter insertion may have devastating long-term sequelae including strictures, incontinence, erectile dysfunction, and infertility. Males are more commonly affected due to their longer urethra. In addition, an enlarged prostate can lead to difficult catheter insertion and a consequently greater likelihood of urethral injury [1]. Research in a single institution in Ireland revealed that of 864 inpatient referrals to a urology department, 6% related to urethral injury resulting from male catheterization by clinicians other than urologists [2]. An American study reported an incidence of 3.2 cases per all 1000 male admissions to a single hospital [3]. Of all urethral injuries recorded by Polish urology units between 1995 and 1999, 32.9% resulted from catheterization [4].

Although progression from medical education to clinical practice differs from country to country, in general junior doctors will spend a year or more after completing medical school as interns or foundation doctors in teaching hospitals developing and demonstrating their clinical competencies before progressing to specialist residency training. It is considered essential for safe patient care that basic practical clinical skills are in place at this early post medical school stage of a doctor's training. For example, recommendations for minimum standards in scholarship, practice and professionalism for those completing medical school published by the UK's General Medical Council include urinary catheterization in male and female patients [5].

Yet authors in at least four continents have questioned whether training in urinary catheterization of junior doctors prior to their regular and close involvement in the care of patients is sufficient [3,6-9]. A study in a large Irish teaching hospital reported that three quarters of catheterization related morbidities occurred when the procedure was performed by interns [2].

With more detailed evidence relating to the insufficiency of training and knowledge in junior doctors it would be possible to design simple educational strategies that can address this knowledge deficit. This study aims to describe the knowledge and experience of interns in a university teaching hospital regarding catheter insertion technique in male patients and regarding urethral injury that can result from traumatic insertion.

## Methods

### Study Design

Questionnaire survey.

### Study Population

All medical interns in Philippine General Hospital, Manila, the national university teaching hospital. All had previously completed at least four years of medical education in an accredited medical school in the country. In the Philippines, urethral catheterization, especially in men, is generally not performed by nursing staff and so training in catheterization is included in the medical curriculum. The curriculum also encourages involvement in patient care during clinical rotations in the final years of medical school, which can include supervised catheterization. The interns were surveyed during the first four months of their medical internship. Internship generally lasts for one year during which the junior doctors develop their clinical skills and experience before taking their medical licensing examinations and progressing to specialized residency training.

### Data collection

A questionnaire was designed for the study to gather data relating to the training, knowledge and experience of medical interns in their first six months of training in a tertiary university hospital on urethral catheterization. The questionnaire contained 17 items covering 4 areas: the manner of acquiring theoretical knowledge and skills on inserting a Foley catheter and level of experience in performing the procedure; perceived adequacy of both theoretical and practical training; theoretical knowledge of the step by step practice of inserting a Foley catheter; the mechanisms of iatrogenic urethral injury arising from improper catheterization (Additional file 1). The elements of the questionnaire that considered perceived adequacy of training were based upon a questionnaire previously developed by Thomas and colleagues [2]. Data collected were limited to factors that can contribute to increased risk of urethral injury rather than wider aspects of catheterization such as aseptic technique. Data were not collected relating to choice of catheter because for indwelling catheterization at Philippine General Hospital only latex Foley catheters are available.

A round table discussion with the consultant urologists in the institution was conducted to evaluate whether the questionnaire adequately addressed elements such as theoretical and practical knowledge relating to correct catheter insertion, methods and adequacy of training and knowledge acquisition, experience of catheter insertion and awareness of the related mechanisms of iatrogenic urethral injury. The questionnaire was then piloted with 10 medical interns who were excluded from the full survey. These interns were asked to comment on the clarity of items on the questionnaire. Modifications on the questionnaire were made based upon this feedback.

The questionnaire was administered from July to August 2010 to all medical interns. Participation in the survey was voluntary and responses were anonymous. The answers to the questionnaires were then collected and results collated and analyzed in Microsoft Excel. The interns were given a short lecture/video presentation on proper insertion of a urethral catheter by the principal investigator at the conclusion of the study.

### Statistical analysis

Descriptive statistics are reported relating to interns' perceptions of training adequacy and knowledge relating to Foley catheter insertion. Chi-squared tests were used to consider associations between self-reported training characteristics (adequacy of theoretical and practical training, training methods received, supervision and confidence) and Foley catheter insertion knowledge and practice (aspects of step by step practice, understanding of adverse scenario). All significant associations are reported. Analysis was conducted using SPSS 16.0 for Mac.

### Ethical approval

The protocol of this study was reviewed and approved by the Philippine General Hospital Expanded Health Research Office ethics review board.

## Results

### Training and experience

Fifteen interns were unable to participate in the survey because of rotation outside the hospital. Of the 240 invited to participate, 225 (94%) completed the survey. 130 (57.8%) were female. At the time of the survey, all of the interns had performed at least 10 catheterizations and 223 (99%) said they were confident with their clinical skills in performing the procedure.

Table 1 reports the perceived adequacy of theoretical and practical training in Foley catheter insertion and the methods of training received. Although 215 (95%) interns who received training said it helped them perform catheterization, only 55.6% of interns responded that they had had adequate theoretical training and 66.7% adequate practical training.

**Table 1:** Methods and interns' perception of adequacy of training on Foley catheter insertion

Perception of adequacy of training			
	Adequate	Minimal	None
Theoretical	125 (55.6%)	81 (36%)	19 (8.4%)
Practical	150 (66.7%)	70 (31.1%)	5 (2.2%)

**Methods of training**			

Demonstration followed by return-demonstration			198 (88.0%)
Lectures			134 (59.6%)
Videos			92 (40.0%)
Reading materials			51 (22.7%)
None			10 (4.4%)
Supervised at first Foley catheterization			204 (90%)

### Reported step-by-step practice of catheterization

Table 2 reports the steps practiced by the interns on Foley catheter insertion. Only 53 (23.6%) reported that prior to performing male urethral catheterization they always take a short history relating to previous catheterization or previous surgery, whilst 101 (44.9%) sometimes do so. Compared with those who reported adequate theoretical training, those who reported minimal or no theoretical training were less likely to take a history (p = 0.009). The taking of a history was positively associated with having received lectures on catheterization as part of their training (p = < 0.01).

**Table 2:** Interns' reported steps in Foley catheter insertion

Taking history on previous catheterization or surgery	
Yes	53 (23.6%)
Sometimes	101 (44.9%)
No	71 (31.6%)

**Lubrication prior to catheterization**	
Lubricant applied to the catheter	220 (97.8%)
Lubricant applied to the meatus	1 (0.4%)
Lubricant injected into urethra	4 (1.8%)

**Angle at which penis is stretched prior to catheterization**	
Parallel to the body	33 (14.7%)
Perpendicular to the body	178 (79.1%)
No particular angle	14 (6.2%)

**Level or depth of catheter insertion**	
Past the mid-point of the shaft of the catheter	24 (10.7%)
To the hub (where the connection for a drainage tube and the inflation port meet)	191 (84.9%)
No particular level: balloon is inflated as soon as urine passes from the catheter	10 (4.4%)

Nearly all (220/225 or 97.1%) apply the lubricant to the catheter, but only 4 (1.8%) inject 10 mL of lubricant into the urethra. Those not trained by demonstration and re-demonstration methods were less likely to lubricate the urethra in line with widely accepted good practice (p = 0.019) [1].

### Knowledge of insertion-related adverse events

Interns were presented with an adverse event scenario in which an elderly male patient is catheterized but no urine has passed from the catheter after two hours. The patient has a distended urinary bladder and is complaining of hypogastric pain. Asked to select the most plausible explanation, 172 (76.4%) interns were able to answer that the catheter was probably not inserted into the bladder. A similar number, 170 (75.6%) interns, entertained the possibility that an iatrogenic urethral injury may have been caused by incorrect catheterization. Asked why blood come from the meatus of the patient upon removal of the catheter, 119 interns (52.9%) were able to answer that urethral injury is likely to have been caused by inflation of the catheter balloon while still in the urethra. Only 64 (28.4%) interns had encountered such a case during their hospital duties. Of the patient factors that were listed that may have contributed to the adverse event scenario, 194 interns (86.2%) identified the presence of urethral stricture and 169 (75.1%) an enlarged prostate as potentially contributing to an increased risk of urethral injury on catheterization. Only 64 (28.4%) identified a long urethra in males, 89 (39.6%) uncooperative patients, and 101 (44.9%) previous catheterization. Of catheter insertion process factors listed, 170 (75.6%) were able to identify inflation of the balloon while it is still in the urethra.

## Discussion

### Summary of main findings

Although the majority of interns participating in this study had performed more catheterizations than those in previous studies and were confident in their skills, there was still a widespread perception that training in catheter insertion is lacking. Gaps exist in the knowledge and practice of interns on the step by step performance of catheter insertion compared to the urologic standard, and a significant number of interns are unable to identify an iatrogenic injury, the mechanism of injury, and factors that can contribute to an increased risk of such injuries. Training in catheterization should be reviewed and redesigned in order to prevent avoidable patient injury.

### Strengths and limitations

This study has considerable strengths in the context of existing literature. All interns in one year in a national government teaching hospital were surveyed. The response rate was very high and the survey includes considerably more interns than previous studies. The questionnaire investigated interns' knowledge in greater detail than previously and the study establishes a baseline for the future evaluation of an educational intervention in the same institution. The study also has limitations that must be acknowledged. The questionnaire was not validated. That said, the methods are comparable with previous research. The survey is set in a teaching hospital in an Asian country and the generalizability of its findings to other regions is uncertain. From a skills training methodology point of view, direct observation is a better method than a questionnaire survey for evaluating acquired competencies, but this was not possible due to resource constraints. It is unfortunate that the questionnaire did not record the proportion of interns who explain the catheterization procedure to patients in order to put them at ease, which is standard practice, as patient anxiety may contribute to difficult catheterization. Lastly, asking multiple-choice questions without feeding the correct answers to the respondent is a challenge. However, the questionnaire was reviewed by senior consultants involved in the development of examinations for medical graduation and was piloted amongst interns.

### The study in the context of the literature

Interns in this study reported high levels of experience of catheterization compared with those in a previous survey of first year interns in the UK published by the Annals of the Royal College of Surgeons of England in 2010, which found that one in five had never performed male catheterization and nearly half (45%) had never performed female catheter [7]. In addition, supervision at first catheterization in this study is higher compared to a previous Irish study [2]. The high level of experience in this study may be born of necessity through internship in a charity hospital in a developing country where catheterization is seldom performed by nurses and where working hours are longer and the patient to clinician ratio is generally much higher than in many other countries.

Nevertheless, the results show that only just over half (55.6%) of medical interns feel that they have had adequate theoretical training and two-thirds (66.7%) adequate practical training. Although better than the 36% and 52% reported by the Irish study that used the same questionnaire [2], these results are still worrying. Similar interns' concerns about the adequacy of their training are echoed in a French study in which only 26% felt that they were able to perform male catheterization and 38.3% female catheterization at the end of their medical education [6].

Although generally experienced and confident in the procedure, shortcomings were identified in the interns' knowledge of correct catheterization practices. It was found that less than a quarter of interns surveyed ask for a history of previous catheterization and surgery, knowledge of which can help predict the presence of urethral strictures or enlarged prostates [1]. Only a handful of interns inject 10 mL of lubricant in the urethra, a practice that ensures adequate lubrication and more efficient insertion and can be of particular benefit in the presence of prostatic enlargement [1]. Although a majority of participants report that they insert the catheter to the correct level before inflating the balloon, it is of concern that more than 10% report inserting the catheter to the shaft only or that they inflate the balloon when urine flows regardless of level inserted, practices that can increase the risk of iatrogenic urethral injury and indicate a training deficit. While three quarters of the interns correctly identified the cause of the catheter-related problem presented to them, only just over half were able to identify the mechanism of an associated iatrogenic injury and overall understanding of possible contributory factors in such injuries was poor.

It has been reported previously that lack of knowledge and skills have led to improper catheterization and urethral injury [2]. Gaps in knowledge and skills were detected amongst interns in this study despite relatively high levels of experience in catheter insertion compared to studies from other countries. It is possible, therefore, that the risks of improper catheterization are higher elsewhere and the need for improved training greater.

### Implications for medical education

More thorough training of incoming medical interns in urinary catheterization may help improve knowledge and practice and reduce the risk of complications and injury. Our study supports previous suggestions that urologists should take the lead in training medical students in urethral catheterization [3,6,7]. Participating interns displayed more experience and confidence than those in other studies, yet gaps in knowledge were identified relating to specific elements of the catheterization process and to factors that can increase the risk of urethral injury. French research highlighted the beneficial effect on trainees' confidence in catheterization associated with a period of training in a urology department [6].

Whether through lectures, video or demonstration, training should be carefully designed to establish the step by step catheterization procedure, with particular emphasis on the key points of lubrication, position of the penis and the extent to which a catheter should be inserted. The importance of history taking prior to the insertion should be stressed and conditions and scenarios that are associated with increased risk of urethral injury upon catheterization should be discussed. The timing of training sessions should also be considered. Given the risk of patient injury associated with the procedure, it may be best to have a session delivered by urologists just prior to the start of internship.

### Implications for research

Training in catheterization is being redesigned in our institute in accordance with the findings of this study, and a further survey of interns in two years will shed light in the effectiveness of these changes. Similar studies in other regions and settings may provide evidence as to the generalizability of our findings.

## Conclusions

Training in catheterization should be universal and should be designed to include step by step instruction in the process, emphasis on history taking and raising of awareness of factors associated with increased risk of urethral injury. Given the risk of serious patient injury, training might best be delivered by urologists just prior to the start of internship when junior doctors commence regular close clinical contact with patients.

## Competing interests

The authors declare that they have no competing interests.

## Authors' contributions

MM contributed to the design of the study, data collection and collation and to the drafting and final preparation of the paper. MCML contributed to the design of the study and to the drafting and final preparation of the paper. BSB conducted data analysis, contributed to the design of the study and to the drafting and final preparation of the paper. All authors read and approved the final manuscript.

## Pre-publication history

The pre-publication history for this paper can be accessed here:

http://www.biomedcentral.com/1472-6920/11/73/prepub

## Supplementary Material

Additional file 1 **Catheterization questionnaire**. The questionnaire used to gather data relating to the training, knowledge and experience of medical interns in their first six months of training in a tertiary university hospital on urethral catheterization. Click here for file
