# Supplementary material for: Medical interns' knowledge and training regarding urethral catheter insertion and insertion-related urethral injury in male patients
Source: BMC Med Educ. 2011 Sep 27;11:73. doi: 10.1186/1472-6920-11-73 (PMC3189902; doi:10.1186/1472-6920-11-73)
Supplement: Additional file 1 — Catheterization questionnaire. The questionnaire used to gather data relating to the training, knowledge and experience of medical interns in their first six months of training in a tertiary university hospital on urethral catheterization. [file 1472-6920-11-73-S1.DOC]

**Appendix A: Urethral catheterization survey**

Kindly answer the following questions honestly and to the best of your knowledge. These are not exam questions. They are survey questions. The questionnaire will take you approximately 5 minutes to answer. Thank you for your participation.

1. Did you receive formal training in urinary catheterization? Circle your answer.

A. Theoretical training

1. None
2. Minimal
3. Adequate

B. Practical training

1. None
2. Minimal
3. Adequate

2. Circle mode/s of learning you had prior to internship regarding urinary catheterization (all applicable).

1. Lectures
2. Demonstration, return demonstration
3. Videos
4. Reading materials
5. None

3. Did these learning activities and materials help you in performing urinary foley catheter insertion?

1. Yes
2. No

4. Have you performed a urinary foley catheterization?

1. Yes
2. No

If yes, proceed to item 5.

If no, proceed to Item 7.

5. How many urinary foley catheters have you inserted so far? (Estimate)

1. Less than 10
2. More than 10

6. Were you supervised during your first urinary foley catheter insertion?

1. Yes
2. No

7. At this point of your medical training, are you confident in your skill in inserting a urethral foley catheter?

1. Yes
2. No

8. Before inserting a catheter, I usually do a quick history on previous catheterization or prior surgery

1. Yes
2. No
3. Sometimes

9. How do you apply the lubricant prior to insertion of a catheter?

1. Apply lubricant to the catheter
2. Apply lubricant to the meatus
3. Inject 10 ml of lubricant into the urethra

10. At what angle do you stretch the penis prior to catheter insertion?

1. Parallel to the body
2. Perpendicular to the body
3. No particular angle

11. In males, up to what level or depth do you insert a foley catheter before inflating the balloon? (Refer to figure 1.)

1. At least past the mid-point of the main shaft of the catheter
2. To the hub (where the connection for a drainage tube and the inflation port meet)
3. No particular level, as soon as urine comes out from the catheter, I immediately inflate the balloon


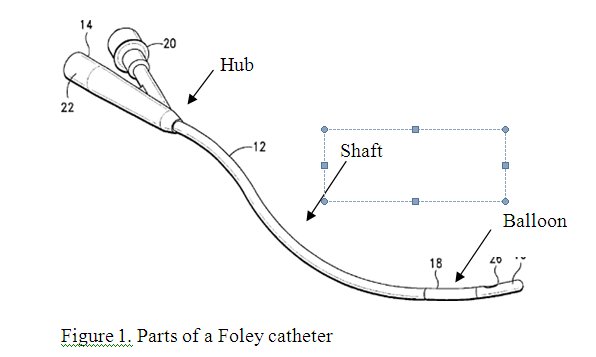


For questions 12 – 17 : After inserting a foley catheter in an elderly male, no urine came out from the catheter after 2 hours. The patient is complaining of hypogastric pain and the urinary bladder is distended on physical examination.

12. What is the most plausible explanation for the patient’s condition?

1. Defective foley catheter
2. Catheter was not properly inserted into the bladder
3. Too much lubricant clogged the catheter
4. Patient has poor urine output

13. Would you entertain an iatrogenic urethral injury as a possibility in this case?

1. Yes
2. No

14. You removed the catheter and blood came out from the meatus. What is the most plausible explanation?

1. Bleeding came from the bladder due to over-distention from urine
2. Bleeding is due to abrasions in the urethra from catheter insertion
3. Bleeding is due to abrasions in the prostate due to an enlarged prostate in an elderly male
4. Bleeding is due to injury to the urethra from inflating the balloon in the urethra

15. Have you encountered a similar case in clinical practice?

1. Yes
2. No

16. Which of the patient characteristics below may contribute to the above scenario? Circle all that apply.

1. Long urethra in males
2. Enlarged prostate
3. Presence of urethral stricture
4. History of previous catheterization
5. Uncooperative patient

17. Which of the practices below is most likely to contribute to the above scenario? Circle the best answer.

1. Lubricating the entire length of the catheter prior to insertion
2. Using the wrong catheter size
3. Inflating the balloon of the catheter when it is inserted midway through the urethra.
